# Edible Insects in Thailand: An Overview of Status, Properties, Processing, and Utilization in the Food Industry

**DOI:** 10.3390/foods12112162

**Published:** 2023-05-26

**Authors:** Sasiprapa Krongdang, Patcharin Phokasem, Karthikeyan Venkatachalam, Narin Charoenphun

**Affiliations:** 1 Faculty of Science and Social Sciences, Burapha University Sakaeo Campus, Sakaeo 27160, Thailand; sasiprapa.kr@buu.ac.th; 2 Bee Protection Laboratory, Department of Biology, Faculty of Science, Chiang Mai University, Chiang Mai 50200, Thailand; patcharin_ph@cmu.ac.th; 3 Faculty of Innovative Agriculture and Fishery Establishment Project, Prince of Songkla University, Surat Thani Campus, Makham Tia, Muang, Surat Thani 84000, Thailand; karthikeyan.v@psu.ac.th; 4 Faculty of Science and Arts, Burapha University Chanthaburi Campus, Chanthaburi 22170, Thailand

**Keywords:** entomophagy, edible insect, gastronomy, nutrition, food process, sustainability

## Abstract

Edible insects have become increasingly popular in Thailand as a nutritious and appealing alternative food source. As the edible insect industry in the country expands rapidly, efforts are being made to transform it into an economically viable sector with substantial commercial potential. Some of the most consumed and sold edible insects in Thailand include locusts, palm weevils, silkworm pupae, bamboo caterpillars, crickets, red ants, and giant water bugs. With its strong growth, Thailand has the potential to emerge as a global leader in the production and promotion of edible insect products. Edible insects are an excellent source of protein, fat, vitamins, and minerals. In particular, crickets and grasshoppers are protein-rich, with the average protein content of edible insects ranging from 35 to 60 g/100 g of dry weight or 10 to 25 g/100 g of fresh weight. This surpasses the protein content of many plant-based sources. However, the hard exoskeleton of insects, which is high in chitin, can make them difficult to digest. In addition to their nutritional value, edible insects contain biologically active compounds that offer various health benefits. These include antibacterial, anti-inflammatory, anti-collagenase, elastase-inhibitory, α-glucosidase-inhibitory, pancreatic lipase-inhibitory, antidiabetic/insulin-like/insulin-like peptide (ApILP), antidiabetic, anti-aging, and immune-enhancing properties. The Thai food industry can process and utilize edible insects in diverse ways, such as low-temperature processing, including refrigeration and freezing, traditional processing techniques, and incorporating insects into products, such as flour, protein, oil, and canned food. This review offers a comprehensive overview of the status, functional properties, processing, and utilization of edible insects in Thailand, and it serves as a valuable resource for those interested in edible insects and provides guidance for their application in various fields.

## 1. Introduction

Insects are among the most abundant creatures globally, with over 2100 species suitable for consumption [1]. Entomophagy, the practice of consuming insects, has been passed down through generations as a local tradition. People learned over time which insects were safe and edible, leading to the development of insect-eating culture. Historically, insect consumption has been primarily limited to rural communities in Thailand, particularly in the northeastern and northern regions, where insects are readily available as a meat substitute. Popular insect foods in Thailand include Cockchafer (Kinun), Dung Beetles, and Giant Water Bugs. Entomophagy remains a common practice in over 100 countries, primarily in Asia, Africa, Oceania, and Latin America [2]. As novel foods, insects have attracted attention worldwide, including in the European Union. Insects can be consumed at all growth stages, from eggs to adults, and their consumption has gained popularity due to their high nutritional value. They are excellent sources of protein, healthy fatty acids, fiber, vitamin B2, and niacin. Certain insects also possess therapeutic properties and may be used as preventive medicine for humans [3], a practice known as entomotherapy.

Edible insects have been consumed for food and medicinal purposes for thousands of years, with their potential benefits recognized even during food scarcity periods [4]. Edible insects are now considered a promising alternative food source with both nutritional and therapeutic benefits. In addition, they offer a solution to the pressing threat of rapid global climate change. Changing human lifestyles is necessary to address this crisis [3]. Since livestock production contributes to over 14.5% of all greenhouse gas emissions, reducing our dependence on livestock for protein and calories is essential. Edible insect farming, or “mini livestock,” generates fewer greenhouse gas emissions, requires less space and water, has shorter life cycles, and has higher feed conversion rates compared to traditional livestock [1,3]. The insect food industry is quickly advancing, developing new products, such as powders, liquids, and oils with various applications [5,6]. Insects can also serve as a sustainable substitute for traditional animal proteins, such as beef, pork, and chicken [7], making them a potential ingredient in future food products.

This review article aims to offer valuable insights into the world of edible insects, focusing on Thailand. It covers topics, such as the chemical composition and biological properties of edible insects, their processing and utilization in the food industry, and the opportunities and challenges associated with their consumption. The information presented will serve as a useful guide for those interested in edible insects and their potential applications in various fields. As the demand for alternative protein sources grows, insects are becoming increasingly popular due to their high nutritional value and promising bioactivities. Insects provide a rich source of quality protein, making them an attractive choice for the food industry. With their potential for multiple applications, insects have a bright future in food and nutrition.

## 2. Thai Edible Insects: Sustainable Prospects and Challenges

### 2.1. Commercial and Edible Insects in Thailand

**Figure 1:**
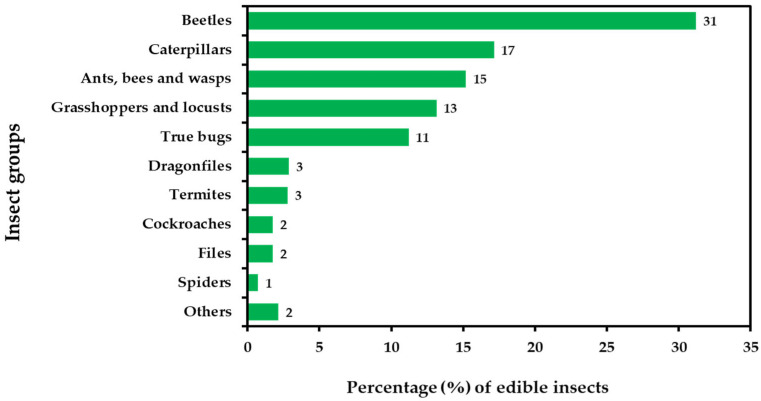
Representatives from almost all edible insect species per group in the world [22,23].

The globalization of the food industry has led to a surge in demand for edible insects. They have long been recognized as a viable protein source in developing countries, contributing to poverty reduction, and fulfilling protein requirements [8]. Edible insects have become a significant income source for rural communities in many Third World countries [9]. Insect consumption is now seen as part of local culture [10] and a source of novel nutrients, such as protein, fat, and other nutrients [11], as well as unique gastronomic experiences [12]. The Asia Pacific region has the most extensive variety of edible species, and with a growing population and increasing food demand, reliance on edible insects is expanding. Countries, such as China, South Korea, Thailand, Burma, Vietnam, and Laos [13,14], are known for consuming and retailing edible insects [15]. In Thailand, entomophagy has been a traditional practice for centuries, primarily in the northern and northeastern regions [16]. Locals have traditional knowledge about edibility, nutrient composition, and preparation methods. Thailand has at least 194 species of edible insects, with over 50 available year-round [17]. Commonly consumed insects include silkworm pupae, bamboo worms, grasshoppers, locusts, cicadas, beetles, crickets, red ants, and others (Figure 1) [17,18,19].

**Figure 2:**
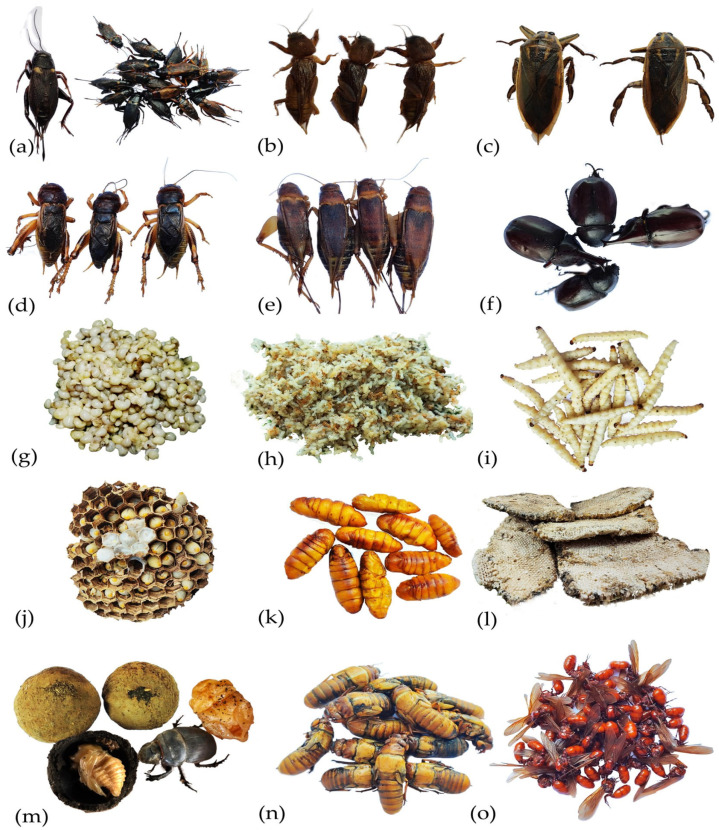
A representative image of fresh and frozen edible insects typically sold in local markets annually, (**a**) African cricket adults (*Gryllus bimaculatus*), (**b**) Mole cricket adults (*Gryllotalpa africana*), (**c**) Giant water bug adults (*Lethocerus indicus*), (**d**) Short-tailed cricket adults *(Brachytrupes portentosus*), (**e**) Ground cricket adults *(Teleogryllus mitratus*), (**f**) Scarab beetle adults *(Xylotrupes gideon*), (**g**) Subterranean ant eggs (*Carebara castanea)*, (**h**) Red ant/weaver ant larvae and pupae *(Oecophylla smaragdina*), (**i**) Bamboo caterpillar larvae (*Omphisa fuscidentalis*), (**j**) Paper wasp larvae and hive *(Vespa affinis*), (**k**) Silkworm pupae (*Bombyx mori*), (**l**) Honey bee larvae and hives (*Apis dorsata*), (**m**) Dung beetle (*Paragymnopleurus aethiops*), (**n**) Cicada (*Meimuna opalifera*), (**o**) Subterranean ant (*Carebara castanea*).

**Figure 3:**
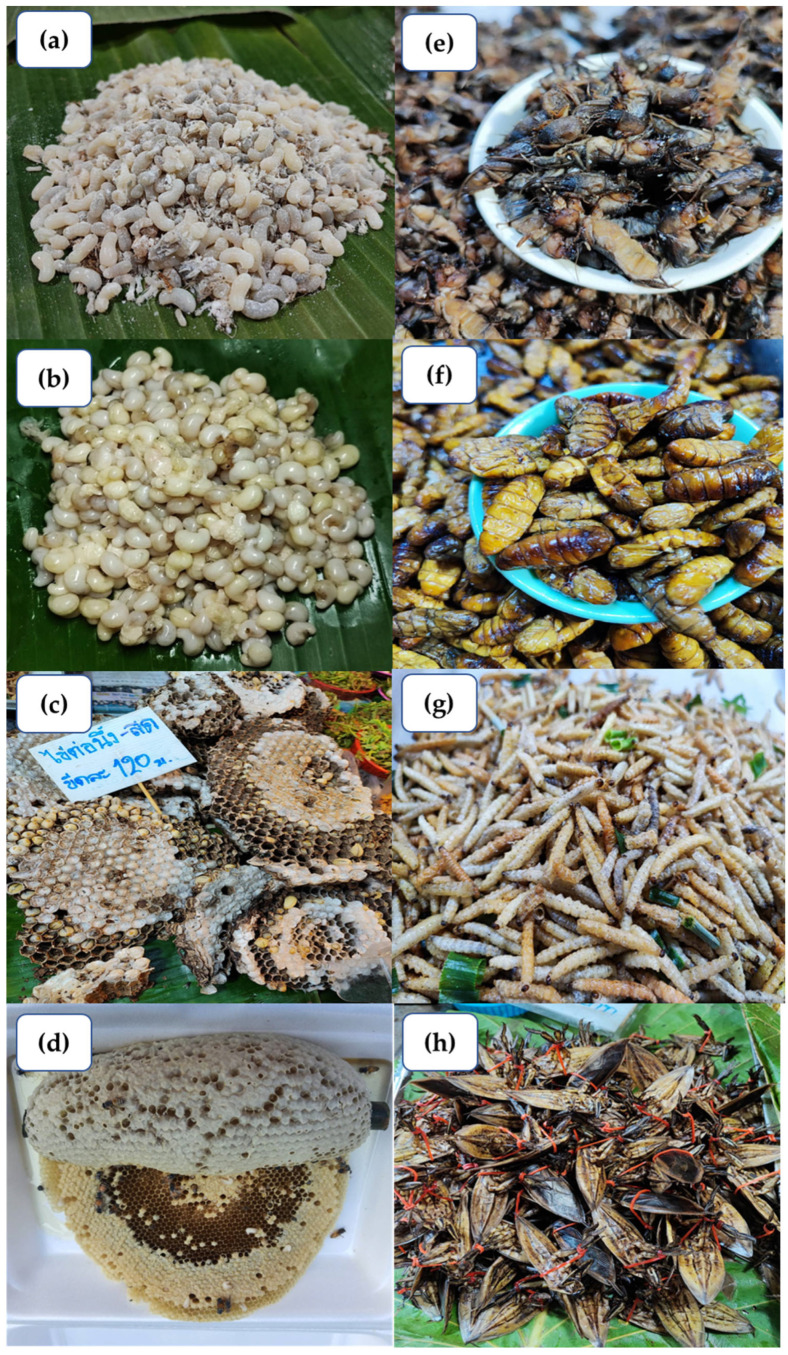
Examples of fresh and cooked edible insects traditionally consumed as food resources in Thailand. (**a**) Fresh red ant/weaver ant larvae and pupae, (**b**) fresh Subterranean ant eggs, (**c**) fresh paper wasp larvae and hive, (**d**) fresh honey bee hive and honey (*Apis florea*), (**e**) fried mole crickets, (**f**) fried silkworm pupae, (**g**) fried bamboo caterpillar larvae, and (**h**) fried giant water bugs.

Thailand’s Ministry of Public Health recommends insect consumption for rural communities to meet their nutritional needs [20], and it has also gained popularity in urban markets [11]. Various cooking recipes are used depending on the insect type, such as roasting crickets and beetles and frying locusts. Insects are also incorporated into dishes, such as chili paste and salads. Table 1 outlines commonly marketed and consumed insect species in Thailand, both seasonally and year-round. Insect consumption trends vary across cultural practices, religions, and geographical regions in Thailand [20]. The diversity of insect foods is more noticeable in urban regions, where people consume insects as a main dish, snack, or both [16]. Several insect food manufacturers in Thailand supply raw materials, such as insect flour, to business partners who produce products, such as pasta, bakery items, ramen noodles, and protein powder supplements for both domestic and global markets [17,21]. Edible insect consumption is widespread in Thailand, with insects available in local markets and in the wild year-round [22]. Insect-based Street food is a popular aspect of Thai cuisine. To ensure consistent availability, some edible insect species, such as crickets, are commercially mass-reared, while other species found in the wild are consumed seasonally (Figure 2). Vendors offer pre-cooked and frozen insect products through traditional markets and online platforms. With growing demand, companies now provide various packaged insect products in dried, frozen, deep-fried, BBQ, and raw forms (Figure 3). These snacks are sold in retail supermarkets, convenience stores, and online platforms across Thailand, promoted as nutritious and ready-to-eat snacks [18,19].

### 2.2. Edible Insect Market Potential and Food Industry

**Figure 4:**
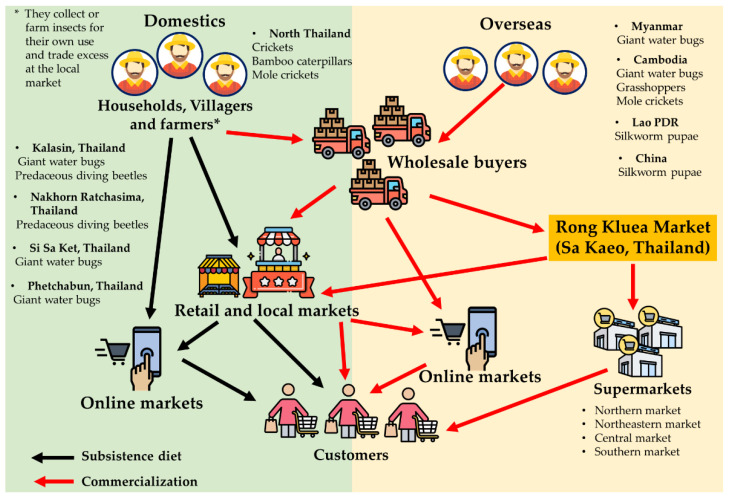
Market and distribution chains of insects in Thailand for subsistence use and the retail sector [16,27,29].

**Figure 5:**
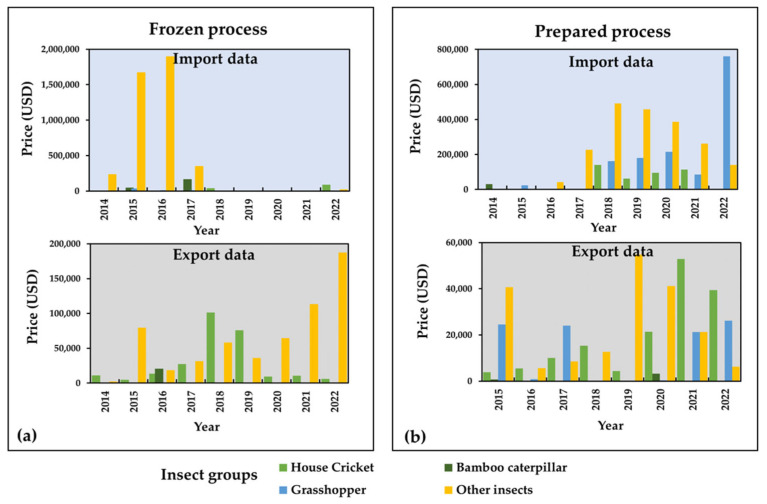
Import and export data on edible insects in 2014–2022; (**a**) frozen edible insect products and (**b**) prepared edible insect products. (Data source: Customs Department of Thailand; https://www.customs.go.th/statistic_report.php; accessed on 12 February 2023) [34].

The edible insect market in the Asia Pacific region is estimated to grow by $270 million by 2024 [8]. In Southeast Asia, countries, such as Thailand, Cambodia, and Laos, have seen a significant increase in insect farming and production volume [14,16]. Thailand has the potential to become a leading producer and provider of edible insect products for the global population [23]. Since 2004, Thailand has been developing top insect-containing products in the Asia Pacific region and holds a 12% world market share for producing edible insect products or protein [23,24]. Insect farming is prevalent throughout Thailand, with the majority of farms located in Northeastern Thailand. It is estimated that 7500 tons of insects, including species collected or farmed in neighboring countries, such as Myanmar, Laos, and Cambodia, are consumed annually in Thailand [3,16,25,26]. The edible insect trade logistics and supply chain overview mainly come from Southeast Asia, particularly Thailand, where well-established farms and trade routes exist [27]. One of the key factors in the edible insect food industry is the lack of systematic work to guarantee safety and shelf-life [24]. Insect farming requires standardization and safety policies, which necessitate government legislation and regulations [28]. Although insect farming has only emerged as a significant economic activity in Thailand in the past two decades, strong market demand, government promotion, university research and extension, and innovative private-sector food processors and sellers have developed production and complied with applicable food safety regulations [8,26]. Insect farming in Thailand can be divided into three types, depending on the harvesting approach: wild harvesting, semi-domestication, and farming [19,29]. More than 20,000 insect farming enterprises are registered in Thailand, with most being small-scale household operations [26]. The dominant insect farmings are the two-spotted cricket (*Gryllus bimaculatus*), followed by the house cricket (*Acheta domesticus*) and ground cricket (*Teleogryllus mitratus*). Presently, 80% of cricket producers are female, and 75% rear on a small scale [30]. Other popular species include the red palm weevil (*Rhynchophorus ferrugineus*), produced by 1000 farmers, and more recently, silkworm pupae have gained interest in the food industry as a byproduct of silk production from the mulberry silkworm industry (produced by 10,000 farmers) [8,31]. Additionally, mealworms and black soldier flies for feed have recently gained traction, with around 100 farmers each producing them [15,16,31,32]. Figure 4 shows the market and distribution chains of insects in Thailand for subsistence use and the retail sector. The supply chain of insects collected by people for subsistence or to be traded or sold in local markets involves traders selling to wholesalers who distribute insects to small retailers or larger supermarkets [29]. This increased commercialization is accompanied by more effective collection techniques, improved transport networks, and the storage of insects in freezers to meet out-of-season demand. Moreover, insects and insect products are increasingly traded online through various social media platforms. As a result, collecting, farming, and marketing insects provide jobs and income for thousands of people in Thailand and neighboring countries. Insects are now consumed globally, with 11 European, 14 Oceania, 23 American, 29 Asian, and 35 African countries all partaking in insect consumption. Among these countries, Mexico, China, Japan, Thailand, and India are the most significant consumers, with the highest number of species consumed [1,33]. In Thailand, the edible insect market has experienced significant growth in recent years, focusing on both export and import flows of products, including frozen and processed goods. From 2014 to 2022, over five species of edible insects have been commercially imported and exported, including crickets, bamboo caterpillars, and grasshoppers, among others [34]. These insect species are primarily imported by China and Myanmar, with a value of over 800,000 USD. On the other hand, the United States, Japan, and England are the primary countries to which these products are exported, with a value of over 200,000 USD in 2022 [34]. Among these insect species, grasshoppers are the most commonly imported, while crickets are the most frequently exported (Figure 5).

According to Thailand’s national strategy (2018–2037), edible insects are being marketed as a sustainable alternative to meet the increasing demand for healthy and nutritious food in both domestic and foreign markets. The farming of insects for economic purposes has the potential to provide secure alternative jobs for farmers. The Department of Agricultural Extension (DOAE) is the primary organization responsible for promoting and developing agricultural jobs related to insects. Farmers in Thailand generate over 7000 million Baht by producing economic insects. The economy and supporting technologies continue to improve the effectiveness of farmers’ insect production. Economic insect farmers can now register with the DAE, allowing the government to maintain a database for precisely planning initiatives to promote and develop production and marketing for farmers or to develop strategies to assist farmers in various domains [27,35,36]. Selling edible insects, whether farmed or wild-collected, can provide economic opportunities for rural communities by diversifying their livelihoods. Although most harvested insects are from the wild, large-scale insect farming for food and feed is still increasing because insects are easy to cultivate and due to growing concerns about the environmental impacts of livestock farming [37]. However, life cycle assessments for insect farming are available for a few species [38]. Moreover, insect farming is generally associated with lower impacts on environmental issues in terms of land and water usage and greenhouse gas emissions than conventional livestock farming, making it attractive from an environmental sustainability perspective [9,27]. European honeybees (*Apis mellifera*), Asian honeybees (*Apis cerana*), stingless bees, lac insects (*Kerria lacca*), and crickets have been officially promoted as economic insects by Thailand’s Department of Agriculture (DOA) [39]. The farming of economic insects has the potential to create direct and indirect jobs, income, and livelihoods along the value chain. This effort is part of a wider strategy to improve peacebuilding, resilience to fragility, conflict, and violence, and create a more stable and sustainable food system that provides economic opportunities using fewer natural resources. The DOA continually supports new innovative agricultural technologies to increase the efficiency of economic insect raising for farmers. Insect farming, particularly farmed cricket, is a new circular food economy that will create climate resilience, jobs, and income and improve nutrition as novel foods that provide high protein widespread throughout the global market [40]. The DOA has transferred knowledge on raising the quality of economic insects to meet market demand and encourages farmers to be certified by Good Agricultural Practice (GAP) standards [39].

In Thailand, GAP has been widely applied, especially in the cricket farming sector. The establishment of GAP standards for cricket farming can be considered a significant milestone in the process of further developing the insect farming industry and gaining access to international markets. The EU and United States, in particular, require higher GAP standards [41]. The European Food Safety Authority (EFSA) and Official Controls Regulation (EU) 2017/625 are primarily considered for export to the European market. However, edible insects are regulated under the Novel Food Regulation (EU) 2015/2283. This regulation covers a variety of products from four edible insects (*Tenebrio molitor*, *Locusta migratoria*, *A. domesticus*, and *Alphitobius diaperinus* larvae), available in categories, such as frozen, paste, dried, and powdered formulations [42].

Insects are more environmentally sustainable than other animal proteins and more nutritious than soybeans [43]. Moreover, the above-mentioned insect features point to the high potentiality of this emerging sector. Insect farming will undoubtedly increase overall agricultural production and raise awareness of the potential of insects, which will contribute to political and marketing decisions. In terms of food security, insects for feed and food purposes can play an essential role in fulfilling the Sustainable Development Goals (SDGs) by 2030 [21,44]. Furthermore, trends towards 2050 predict a steady population increase to 9 billion people, which significantly requires food and feed supplies from available agro-ecosystems. This highlights the importance of the environmental sustainability of insect farming compared to livestock production [21].

### 2.3. New Gastronomic Trends

The consumption of insects as food, or entomophagy, is gaining significance in gastronomic culture in more than 113 countries across Asia, Africa, and South America [21,45]. It is also part of modern food trends, alongside other foods, such as algae, sprouts, microbes, edible flowers, and whole grains [46]. The incorporation of edible insects into food as ingredients not only allows for creativity, tradition, and new techniques but also offers a historical perspective on human nutrition and the development of new technologies [47]. Crucial factors in insect production include sensory attributes, protein content, quality, and safety, all of which affect their acceptance by consumers. Edible insect-based dishes adapted to Western sensorial and textural preferences are now available in the market, and insect consumption is gradually transitioning from being a gastronomic experience to a part of daily diets, as exemplified by cricket flour [48]. To increase insect consumption, food aesthetics must become more appealing to consumers. However, integrating edible insects into the Western diet remains a challenge that requires taste education and sharing information about entomophagy. Familiarizing consumers with the taste and texture of edible insects through tastings and overcoming their fear of consuming insects is essential [45].

## 3. Chemical Composition and Important Biological Activities

### 3.1. Chemical and Nutritional Composition

The chemical and nutritional content of edible insects varies based on various factors, such as the species, sex, raising environment, and developmental stages. Nevertheless, insects are generally abundant in nutrients, with protein being the most substantial component, followed by fat, vitamins, and minerals (Table 2).

Crickets and grasshoppers, for instance, are particularly rich in protein, with an average protein content of 35–60 g/100 g of dry weight or 10–25 g/100 g of fresh weight, which is higher than plant protein sources, such as cereals, soybeans, and beans. Insects offer more protein than meat, chicken, or eggs, although their exoskeleton, which is relatively hard and contains high levels of chitin, makes them difficult to digest. Removal of the exoskeleton can improve digestion, with rates as high as 77–98% [3,11]. Chitin is a carbohydrate polysaccharide similar in structure to cellulose, attached to N-acetyl-D-glucosamine by β-1,4 glycosidic bonds, with the hydroxyl (-OH) group at the C2 position replaced by the acetyl amino group (-NHCOCH_3_). Chitin is a component of the insect cell wall and the primary source of fiber in edible insects. However, chitin may reduce the bioavailability of nutrients and protein digestibility [37]. Furthermore, antinutrient content in formulations may have a negative impact on the bioavailability of nutrients, such as iron, zinc, and calcium, which affect the nutritional status of children [53]. For example, Mekuria et al. [54] reported higher levels of tannins and phytates (mg/100 g) in plant-based porridge (208.9 mg and 68.2 mg) than in bee larva-based porridge (119.4 mg and 13.1 mg). The essential amino acid content in insects is high, except for methionine and tryptophan, which are present in low levels. The variation in amino acid content in different species of edible insects can be attributed to various factors, such as the culture system. Among all amino acids, glutamic acid has been found to have the highest content, followed by leucine and lysine [25,55]. The amount of fat in insects is influenced by factors, such as species, sex, reproductive stage, season, food, and habitat. Larvae and pupae contain more fat than adult insects, and females have more fat than males. Insects generally have a higher content of unsaturated fatty acids (UFAs) than saturated fatty acids (SFAs) [3,11]. Palmitic acid and stearic acid are the most saturated fatty acids found in edible insects, while oleic acid is the most abundant unsaturated fatty acid [25,55]. Grasshoppers, crickets, termites, and worms are examples of insects that are rich in iron, zinc, calcium, copper, phosphorus, magnesium, and manganese. Invertebrates without mineralized skeletons have very little calcium content. Insect consumption can provide a high proportion of the daily recommended minerals for humans, particularly iron. Most edible insects also contain vitamins, such as carotene, vitamins B1, B2, B6, C, D, E, and K [3,11].

### 3.2. Biological Activities

In China, the practice of consuming insects has been a part of the country’s culture for thousands of years. Presently, edible insects are gaining popularity as a sustainable and nutritious alternative source of protein owing to their lower ecological impact and high nutritional value [56]. However, the appeal of edible insects lies in the bioactive compounds they contain that may induce functional effects in the body [57]. Despite this, edible insects have been largely unexplored as sources of natural antioxidants [58]. The potential health benefits of edible insects include alleviating oxidative stress-induced diseases and serving as natural antioxidants in the food industry [58]. Some edible insects, including the silkworm (*Bombyx mori*), contain flavonoids, such as quercetin, found in mulberry leaves that influence the quality and yield of silkworm cocooning [59]. Certain phenolic compounds present in insect cuticles, such as catechin, epicatechin, protocatechuic acid, protocatechualdehyde, and 4-dihydroxyacetophenone, and those found widely in the plant kingdom, such as gallic acid, ferulic acid, quercetin, and resveratrol, have been studied [58]. Resveratrol, a phytochemical, has been identified as the main natural antioxidant in some plants [58]. Researchers have found that aqueous extracts and aqueous ethanol have better antioxidant activity [58,60]. Conversely, other aqueous extracts may contain interesting substances, including fatty acids, particularly unsaturated fatty acids (USFAs), such as α-linolenic acid (ALA), which show β-hexosaminidase-inhibitory activity based on aqueous hexane extracts of *Oxya yezoensis* [61] and exhibit dominant inhibitory activity against lipid peroxidation in *Omphisa fuscidentalis*, *Euconocephalus* sp., *Patanga succincta*, *A. domesticus*, and *Lethocerus indicus* [60]. In this section, we present the structures of potential phytochemical substances listed in the literature. Edible insects have been found to exhibit various activities, such as antihypertensive, antitumor, and anti-inflammatory activities, and some species contain potential compounds, such as peptides with antimicrobial and antifungal activity. Although limited information is available on sterol compounds in edible insects that exhibit antidiabetic activity [58], some interesting research has been conducted on the presence of phytosterols, such as campesterol and β-sitosterol, in *T. molitor*, which have been shown to decrease LDL cholesterol levels, potentially lowering the risk of heart disease [62]. Some of these significant findings have been applied or sold as health supplements or functional foods and are summarized in Table 3.

## 4. Processing and Utilization in the Food Industry

### 4.1. Low-Temperature Processing (Refrigeration and Freezing)

**Figure 6:**
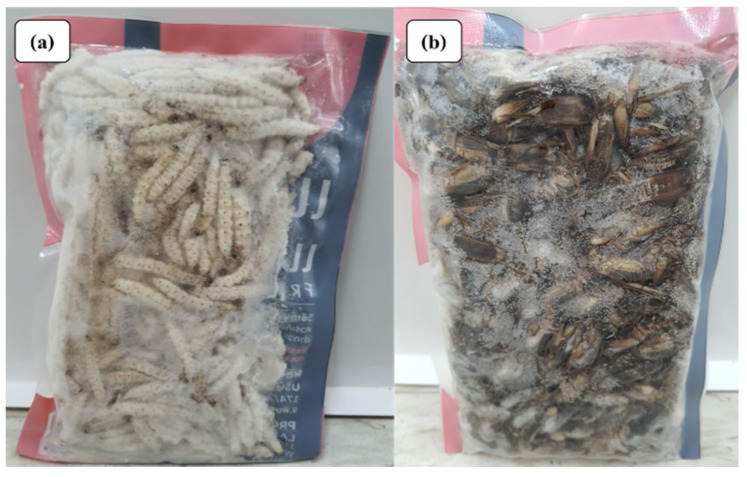
Frozen insects in Thailand markets: (**a**) bamboo caterpillar and (**b**) cricket.

The post-harvest storage of edible insects, intended for purposes other than live sale, typically involves low-temperature storage (Figure 6) [10]. Insect preparation prior to processing is a critical step that can significantly impact the quality of the final product. The choice of life stage the insect to be consumed is an important consideration and can include eggs, larvae, pupae, adults, and post-harvest specimens. Insects are separated from their substrate, sorted by quality, and then culled. In the case of adult insects, parts of the wings, legs, and non-edible waste are removed and cleaned. Subsequently, microbial populations are reduced through scalding in boiling water or steaming, a critical processing step that can impact the final product’s quality, microbial content, chemical composition, color, and flavor [10,90]. Low-temperature insect preservation techniques, such as cooling and freezing, are utilized to extend the product’s shelf life. Low-temperature storage helps to maintain the quality of the insects, with their appearance and organoleptic characteristics remaining unchanged. Freezing, a technique used to lower the temperature of food to below freezing, allows the food’s center temperature to reach −18 °C or lower, causing the water in the liquid food to freeze, reducing water activity, and limiting the number or growth of microorganisms. Freezing slows the spoilage of food and is typically accomplished via two methods: (1) slow freezing, which takes longer than 12 h to freeze, resulting in large extracellular ice crystals when the extracellular solute concentration is high, leading to cellular shrinkage due to intracellular water loss; and (2) rapid freezing, achieved in less than 2 h, which produces small and uniform ice crystals, with cells diffusing both inside and outside the cell and without cellular shrinkage. Rapid refrigeration typically has a minimal impact on food quality, and when defrosted, the food returns to its pre-frozen state [11]. Freezing typically extends the shelf life of insect products more effectively than refrigeration, which involves chilling food to a low temperature to preserve its freshness or processed food to extend its shelf life beyond ambient temperature. De Smet et al. [91] found that spoilage bacteria continued to grow in mealworm paste stored at 4 °C and spoiled after storage for as little as two weeks, while specimens stored at −21 °C remained unspoiled for up to three months.

### 4.2. Traditional Processing Techniques

Edible insects are collected or harvested and sent to food and feed factories as raw materials for processing into value-added products for commercial use. Achieving a high-quality product requires attention to techniques and practices at every stage, from collection or harvesting to primary processing, packaging, storage, and transport to the consumer. Traditional processing methods for edible insects include toasting, boiling, smoking, frying, and roasting. Toasting involves heating live insects in a hot, non-frying pan after cleaning them for approximately 5 min while being turned regularly using a cooking stick to prevent sticking or burning. Boiling involves placing insects in a mesh sieve and immersing them in boiling water at 96 °C for 5 min before removing the sieve from the water. Smoking employs smoke from combustion and heat to allow the product to ripen, dry, and acquire a smoky flavor. Smoking also preserves the product by reducing the pH and preventing the growth of microorganisms on the surface of the insects. For smoking, insects are first cleaned, any unwanted parts are removed, and they are then washed, pickled in brine for approximately 30 min, drained, sprinkled with sugar, spices, or seasonings, steamed, and then smoked at around 50–55 °C for about an hour. Frying uses vegetable oil or animal fat as a heat exchange medium and involves heating the oil to a temperature of 170–210 °C. Pan-frying involves using a small amount of oil or fat to prevent the food from sticking to the frying pan, while deep-fat frying uses a larger amount of oil to fully submerge the food resulting in a dry, crisp, brown exterior. Insects are fried in a skillet and may be turned upside down during the process to ensure even cooking and achieve the desired flavor. Roasting involves heating insects in a pan over an electric stove at around 130 °C for 25 min, and they can be seasoned as desired before being stored in tightly sealed containers at a cool temperature. Freezing insects before roasting and frying significantly increases the fat, protein, ash, energy value, and free fatty acid value and reduces the peroxide value and TBARS value when compared to raw insects [19,92].

### 4.3. Edible Insect Powder

Drying or dehydrating is a well-established method of food preservation. By evaporating most of the water in food through methods, such as dehydration or sublimation, the moisture content is significantly reduced. Processing insects into dry powder extends their shelf life, as drying removes water that can facilitate the growth of various microorganisms, such as mold, yeast, and bacteria, which can cause spoilage and foodborne illnesses. Drying also inhibits enzyme activity and slows down reactions that can contribute to spoilage. By reducing the weight and volume, drying makes food easier to transport, consume, and use as raw materials for further processing. It is an alternative method to transform insects into various food ingredients, making them more palatable and suitable for people who are averse to insect appearances, unfamiliar flavors, and textures. Insect powder is an example of such an ingredient that can offer consumers a wider range of food choices. The process of manufacturing insect powder involves starting with fresh insects and subjecting them to quality selection, washing, blanching, and drying before grinding them into a fine powder. Cricket powder, which is rich in quality protein, is a highly suitable ingredient for the production of bread products. Research has found that increasing the amount of cricket powder enhances the nutritional value of bread, reduces the hardness of the bread, and improves its consistency [7,93]. In a study by Zielinska et al. [94,95], using edible insect starch as an ingredient in muffins was found to have a positive effect on the product’s quality. Increasing the amount of insect starch reduced the brightness of the muffins but improved their texture, making them softer than regular muffins. The addition of insect starch also decreased the amount of rapidly digested starch (RDS) and slowly digested starch (SDS), affecting the glycemic index (GI) value. The GI measures the quality of carbohydrate foods and how they affect blood glucose levels. Foods with a higher GI value cause a greater increase in blood sugar levels. Muffins with added insect starch had lower GI values compared to conventional muffins, making them a suitable option for those who need to avoid high-sugar and high-fat diets due to conditions, such as obesity, diabetes, and insulin resistance.

### 4.4. Edible Insect Protein

The functional properties of insect proteins, such as emulsification, foaming, gelling, and dissolution, are affected by the interaction between chitin and proteins in powder form, which limits their use in food products (Table 4). Guidelines have been developed to extract edible insect protein powder by separating chitin from protein using various methods, including heat, enzyme use, and acids or alkalis. Protein extraction through different methods increases protein content, protein particle size, amino acid sequence, and different functional properties [4,96]. Proteins extracted from insects can be classified into three types: concentrated protein, isolated protein, and protein hydrolysate. Concentrated protein is separated from other constituents, such as sugar and fat, resulting in a protein concentration of about 25 to 89%. Isolated protein is produced from the purification of concentrated protein using different processes, resulting in a protein concentration of 90 to 97% with smaller protein particles. Protein hydrolysate is a product derived from the hydrolysis of proteins by enzymes, proteinases, acids, or alkalis, resulting in smaller peptides or free amino acids. Protein hydrolysate is mainly used to reduce allergies to proteins in food [96]. The protein content of crickets is in the range of 17.51–22.20 g/100 g dry weight, with a protein sedimentation point of pH 5.5. The temperature and time required for extraction affect the amount of protein extracted from crickets, with differences observed between species, diets, and life cycles. However, if the protein is extracted under extreme conditions, it will affect the amino acid structure. It converts it from an L-form to the D-form of amino acids, which the body cannot utilize and is not suitable for use as an ingredient in food products. The easiest, lowest-cost, and lowest-residue method for protein concentrate extraction is the use of temperature and time, which can be applied to communities and small industries [97]. Protein hydrolysate from mealworm, cricket, and silkworm was enzymatically extracted from commercial enzymes. The solubility of hydrolyzed proteins was higher than that of unhydrolyzed proteins, with Alcalase-hydrolyzed cricket protein hydrolysate showing the best emulsifying properties due to its high emulsion stability. Insect protein hydrolysate has an inhibitory effect on ACE activity and α-glucosidase, with the Alcalase-digested cricket protein having the greatest inhibitory effect on ACE activity. Silkworm proteins showed anti-inflammatory activity. The chitin structure in edible insects can be degraded by acid. The protein sludge derived from acidic digestion can be used as an ingredient in various food products, such as sausages and processed meat products [5,6,82,98]. High-pressure processing is a food preservation technique that does not use heat. High hydrostatic pressure (HHP) at 70–600 MPa for 5 min causes the aggregation of high molecular weight proteins and an increase in the hydrophobicity of denatured proteins, affecting the functional properties of proteins extracted from mealworm. High-pressure applications ranging from 100 MPa to 800 MPa, and sometimes up to 1000 MPa, result in non-covalent bond instability. High hydrostatic pressure does not affect the covalent bonds of proteins but causes the exchange of disulfide bonds, especially when proteins with free thiol are pressed under high pressure [20,99].

### 4.5. Edible Insect Oil Extraction

Insects are a rich source of oil, second only to protein. However, to obtain a high protein content, it is necessary to remove the fat during the extraction process. Therefore, utilizing protein by-products through lipid extraction is an attractive option for adding value to these by-products. There are several methods for extracting oil, including solvent extraction with commonly used solvents, such as methanol and ethanol, mechanical extraction, such as compression, and extraction using advanced technologies, such as supercritical fluid extraction. This method involves pushing fluids above their critical points, allowing them to permeate solids, including gases and dissolve solutes, such as liquids. An example of a substance used in this method is supercritical carbon dioxide, which is a gas–liquid mixture that can dissolve non-polar compounds. This method is safer for the environment and consumers compared to the organic solvent extraction method, which may not be able to completely separate the solvent, leading to residual solvent in the final product. A study compared the efficacy of lipid extraction as a by-product of protein extraction in house crickets (*A. domesticus*) and mealworms (*T. molitor*) using various methods, including Soxhlet continuous extraction with solvents, such as hexane, petroleum ether, ethyl acetate, and 95% ethanol, as well as the three-phase partitioning method and supercritical carbon dioxide extraction. The study found that ethanol extraction yielded the highest oil yield (22.7–28.8%). However, in order to increase the efficiency of the extraction process, a combination of methods, such as using a solvent in combination with supercritical fluid extraction, may be necessary [12,100].

**Table 4:** Edible insect processing for food ingredients and food products.

Edible Insect	Processing	Products	Effect of Edible Insect Powder	References
Cricket	Powder drying	Bread	Bread’s structure was improved, and its hardness was decreased as a result of using cricket powder. The health-promoting properties of bread products was reported.	[93]
Mealworms and crickets	Powder drying	Muffin	Increasing the amount of insect powder decreased the lightness. Moreover, it gave a softer texture and a decrease in the hardness, springiness, resilience, cohesiveness, and chewiness of the muffins.	[94]
Cricket	Powder drying	Baked chips	The baked chips revealed a higher quantity of protein, iron, and calcium when compared to the control.	[101]
Cricket	Powder drying	Cookies	The potential to increase protein content in cookies by using cricket powder. As the quantity of cricket powder used in the formulation increased, the organoleptic acceptability of cookies enriched with cricket powder decreased.	[102]
Mealworm	High hydrostatic pressure	Protein	It was discovered that large protein aggregates formed, primarily composed of hexamerin 2 and amylase.	[99]
Giant water bug	Solvent Extraction	Essential flavor	The majority of volatile chemicals were created by the oxidation of lipids. Compounds from the Maillard reaction are produced thermally, such as 2-acetyl-1-pyrroline and 2-acetyl-2-thiazoline. The contribution of the two most prevalent volatiles, (E)-2-hexenyl acetate and (E)-2-hexenyl butanoate, to the overall aroma of the bugs was highest.	[103]
Yellow mealworm, lesser mealworm, house cricket and Dubia cockroach	Aqueous oil extraction	Oil	Except for the Dubia cockroach, these bug oils contained compounds that were linked to pleasant scents. In the latter oil, several acid compounds linked to unpleasant aromas were identified. The yellow meal worm oil, lesser meal worm oil, and cricket oil all have qualities that make them suitable for use as table oils and food ingredients.	[104]

### 4.6. Canned Edible Insects 

**Figure 7:**
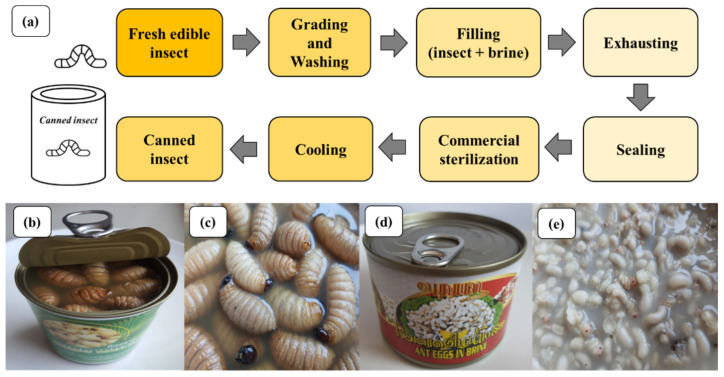
Example of canned edible insects in Thailand markets: (**a**) processing of canned insect in brine, (**b**,**c**) sago worms, and (**d**,**e**) ant eggs in brine.

Edible insects can be canned through a process of thermal processing, which involves placing the insects in sealed containers before commercial sterilization. This allows them to be stored for 6 months to 3 years without deterioration. Commonly canned insects include crickets, coconut weevils, and red ant eggs (see Figure 7). To prepare the raw material, the insects are first quality checked, and unwanted parts are removed. They are then washed and blanched in boiling water for 1–4 min, depending on the nature of the raw materials. A 5% concentration brine is prepared, and the cans are not fully packed to allow for expansion of the liquid during heating. Air is exhausted to create a vacuum condition that inhibits the growth of microorganisms and prevents chemical reactions that can cause changes in color, smell, and taste. This is achieved by spraying hot steam onto the surface of the food with an exhaust. The lid is then closed to create an airtight space above the can, or air may be exhausted before sealing. Thermal sterilization is then used to kill microorganisms, which involves heating the cans at 120 °C for 16 min. After sterilization, the product must be cooled immediately to prevent thermophilic bacteria from flourishing. Canned edible insects can be stored for up to 2 years at room temperature.

### 4.7. Chitin Extraction

**Figure 8:**
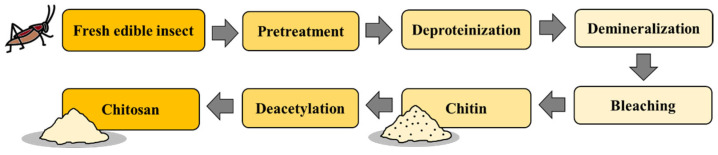
The extraction of chitin and chitosan from edible insects.

Chitin is a biopolymer present in the outer shell of insects, and it is considered a bio-based material that is biodegradable, safe for human use, and environmentally friendly. It has various applications in the food industry, such as a dietary supplement to reduce fat and cholesterol, a stabilizer, thickening agent, and preservative to prevent bacterial and mold growth. Chitin is also used in wastewater treatment as a coagulant, in fruit and vegetable coatings to extend shelf life, and in the clarification of fruit and vegetable juices. Additionally, chitin is useful in protein enzyme production, food technology, biotechnology, biochemical engineering, and more [1,105]. The extraction of chitin involves several processes. First, the protein-removal process (deproteinization) is carried out before or after demineralization. Demineralization is performed by reacting the raw material with alkaline solutions, usually sodium hydroxide, which removes most of the protein along with some fat and pigments. Second, demineralization is carried out through an acid reaction, primarily using hydrochloric acid, which converts most minerals, such as calcium carbonate, to carbon dioxide, along with some pigment and dissolved protein. The resulting acid product from these two steps is chitin. Deacetylation is then performed to convert the acetamido group (-NHCOCH_3_) into an amino group (-NH_2_), which can be achieved by reacting it with concentrated alkali or by enzymatic reaction (Figure 8). Chitosan is obtained when more than 60% of the acetyl group content is eliminated from chitin. Another step-in chitin processing is reducing the particle size, which can be achieved through acidic and enzymatic hydrolysis, as well as the use of oxidizing agents, such as hydrogen peroxide, alkali digestion, and ultrasonic waves [16,106]. Chitin extracted from the beetle *Holotrichia parallela* Motschulsky using 1 M HCl and 1 M NaOH, followed by a potassium permanganate solution at 1% concentration for decolorization, yielded 15% chitin. It showed a similar chemical structure and physicochemical properties to commercial chitin extracted from shrimp [17,107]. Insect chitin from six different species was compared with chitin extracted from shrimp shells and found to have similar chemical and physiological properties, making it suitable for chitosan production. The characteristics of chitin are specific to each insect species, making it useful as a taxonomic tool to diagnose and show the relationship between insect species [108].

### 4.8. Edible Insect Products and Other Uses

Edible insects have diverse uses in numerous industries. The by-products generated during insect processing can be utilized as animal feed ingredients. Buprestidae beetle wings can be fashioned into exquisite jewelry pieces, while giant water bugs are also useful for flavor extraction in food products. Silkworm cocoons, in addition to being used for weaving silk, contain important substances. They are composed of 70–80% fibroin protein and 20–30% sericin protein, and two fibroin fibers are coated in sericin glue. The glue is eliminated during degumming, leading to the production of two types of silk powder: sericin and fibroin. Silk powder is known for its moisturizing and whitening properties and is often used in cosmetics and health drinks [24,109]. Honey is a sweet, syrupy substance produced by bees through the collection and processing of flower nectar or plant parts in their honeycomb. It ranges in color from light yellow to dark brown. Honey has been used for medicinal purposes in many countries because of its ability to treat various ailments, reduce inflammation, and improve skin conditions. It is also an effective natural sweetener that can quickly nourish and refresh the body. Besides its sweet taste, honey contains vitamins, minerals, and antioxidants. Its composition can differ based on factors, such as the source of the nectar, the environment, season, and production process. Nowadays, honey is widely used in various food products as an ingredient [1].

## 5. Conclusions

Many edible insects are used as raw materials for food production in Thailand. Edible insects are rich in nutrition and chemical composition, such as protein, fat, dietary fiber, carbohydrate, minerals, and bioactive compounds. The appearance of edible insects may not be attractive for consumers. Nowadays, insects are processed into a variety of food products in order to improve their appearance, safety and convenience for consumers. The practice of entomophagy, or the consumption of insects, is gaining popularity and is being touted as the “food of the future.” This is because edible insects are known to have a high protein content, and they provide all essential amino acids required for a healthy diet. As the world’s population continues to grow, edible insects represent a promising alternative protein source. However, it is important to note that this field of research is still in its infancy in many countries that have a tradition of entomophagy. Moreover, despite the vast diversity of insect species found worldwide, only a few have been intensively studied and applied in foods. Therefore, it is important to further explore the potential of edible insects in food industries and expand our knowledge of the various species that could be utilized in this manner. The information obtained from this article can be used as a guideline for the further development of studies on edible insects aimed at utilizing the full potential of various fields in the future.

## Figures and Tables

**Table 1:** Edible insects that are commonly and seasonally marketed and consumed in Thailand.

Common Name (English)	Local Name (Thai to English) ^a^	Scientific Name	Edible Stage *	Harvested Source **	Price/kg (USD) ^b^
Bombay locust	Tuck-tan (Patanga)	*Patanga succincta* (Johannson, 1763)	A	W	6.47–7.35
Small rice grasshopper	Tuck-tan (lek)	*Oxya japonica japonica* (Thunberg, 1815) *Locusta migratoria manilensis* (Meyen, 1835)	A	W	5.88–7.35
Yellow-backed grasshopper	Tuck-tan (yai)	*Cyrtacanthacris tatarica* (Linnaeus, 1758)	A	W	5.88–7.35
Cockchafer scarab beetle/June beetle	Ki-noon	*Holotrichia* sp.	A	W/F	5.88–10.30
Red palm weevil/Sago palm weevil	Duang Saku	*Rhynchophorus ferrugineus* (Olivier, 1790)	L	F	7.35–8.82
Dung beetle	Kud-jee	*Paragymnopleurus aethiops* (Sharp, 1875)	P/A	W	0.30–1.50/each
Paper wasps/Lesser banded hornet	Tdaw	*Vespa affinis* (Linnaeus, 1764)	L/P	W/S	44.12–58.82
Silkworm	Dug-dae Mai	*Bombyx mori* (Linnaeus, 1758)	P	F	5.88–14.40
Bamboo caterpillar	Non Mai-pai	*Omphisa fuscidentalis* (Hampson, 1896)	L	W	35.30–73.53
Subterranean ant	Mangmun	*Carebara castanea* (Smith, 1858)	A/E	W	35.30–58.82 (adults) 58.82–73.53 (eggs)
True water beetle	Tub-toa	*Cybister limbatus* (Fabricius, 1775)	A	W	9.70–11.17
Water scavenger beetle	Malaeng niang	*Hydrous cavistanum*	A	W	n.d.
Crickets/African cricket/Mediterranean field cricket/Two-spotted cricket	Jing-reed (Tong dam)	*Gryllus bimaculatus* (De Geer, 1773)	A	W/F	2.94–3.53
Ground cricket	Jing-reed Tongdang	*Teleogryllus mitratus* (Burmeister, 1838)	A	W/F	2.94–3.53
House cricket	Jing-reed (Tong dang li) (sa-ding)/Ji-reed Ban	*Acheta domesticus* (Linnaeus, 1758)	A	W/F	3.82–4.41
Short-tailed cricket	Ji-pome, Ji-koong	*Brachytrupes portentosus* (Lichtenstein, 1796)	A	W/F	3.53
Mole cricket	Kra-chon/Ji-zon	*Gryllotalpa africana* (Palisot de Beauvois, 1820)	A	W/F	4.41
Red ant/weaver ant	Mod-dang Kai-mod-dang Ma-peng	*Oecophylla smaragdina* (Fabricius, 1775)	E/P	W/S	14.70–29.14
Buprestis beetle	Malaeng Tub	*Sternocera aequisignata* (Saunders, 1866)	A	W/S	n.d.
Honey bee	Pung	*Apis* spp.	L/P	W/F	11.76–17.65
Cicada	Juk-jan	*Meimuna opalifera* (Walker, 1850)	A	W	11.76–14.70
Winged Termite (Alates)	Mang-moa	*Termes* sp.	A	W	4.41–5.88
Giant water bug	Mang da na	*Lethocerus indicus* (Lepeletier et Serville, 1775)	A	W/F	Male 0.29–0.58/each Female 0.23–0.50/each
Water scorpion	Mang pong nam	*Laccotrephes ruber* (Linnaeus, 1764)	A	W	n.d.
Scarab beetle/Siamese rhinoceros beetle/Fighting beetle/Rhinoceros beetles	Duang Kwang	*Xylotrupes gideon* (Linnaeus, 1767)	A	W/F	11.76–14.70
Mealworm	Non Nok	*Tenebrio molitor* (Linnaeus, 1758)	L	F	7.35–8.82
Common skimmer/River skimmer	Mang ra ngum	*Crocothemis* sp.	N	W	4.41–5.88

**^a^** Local Thai name of edible insects is listed based on Hanboonsong et al. (2013) [16]. **^b^** Source: Interview from the seller in fresh market and price advertising on the market online in Thailand. Abbreviation: n.d.; there is no data available. * Edible Stage: adult (A), egg (E), larvae (L), naiad (N), and pupae (P). ** Harvested source: farmed (F), semi (S), and wild (W).

**Table 2:** Example chemical composition of edible insects in Thailand.

Chemical Composition	Content			
	Cricket (g/100 g) [49]	Honeybee Larvae (g/100 g) [50]	Giant Water Bug (g/100 g) [51]	Sago Worms (g/100 g) [52]
Protein	63.3	35.3	53.11	10.39
Fat	10.3	14.5	8.15	17.17
Fiber	5.2	-	12.23	-
Ash	5.6	4.1	-	-
Carbohydrate	-	46.1	19.74	-
Minerals	-	-	6.75	-
Calcium	10.1	0.0849	-	0.0149
Phosphorous	7.9	0.7825	-	0.1023
Magnesium	1.2	0.1770	-	0.0526
Zinc	0.215	0.0116	-	0.0029
Copper	0.015	0.0036	-	0.0004
Manganese	0.040	0.0012	-	0.0004
Iron	0.116	0.0131	-	0.0008
Sodium	-	0.0594	-	0.0166
Potassium	-	1.8719	-	0.2046

**Table 3:** Biological properties, bioactive compounds, and species of edible insects that have been reported in Thailand.

Bioactive Compounds	Species	References
Anti-oxidant activity		
Phenolic compounds	*Acheta domesticus*, *Tenebrio molitor*	[62]
4-Hydroxybenzoic acid, *p*-coumaric acid, ferulic acid, syringic acid	*Acheta domesticus*	[63]
Catechin (phenolic compounds) and proteins	*Holotrichia parallela*	[58]
Peptides (CTKKHKPNC)	*Oecophylla smaragdina*	[64]
Total phenolic compounds	*Rhynchophorus ferrugineus*	[65]
Total phenolic compounds	*Tenebrio molitor*	[66]
Crude extract	*Vespa affinis*	[67]
Total phenolic compounds, sericin proteins (silk cocoon), Silkworm pupae protein concentrate, 35-kDa protein, 1-deoxynojirimycin (DNJ)	*Bombyx mori*	[68,69]
Crude extract	*Teleogryllus emma*	[70]
Total phenolic contents	*Oecophylla smaragdina*	[71]
Unknown	*Apis mellifera* (bee tea)	[72]
Total phenolic acids and flavonoids	*Apis mellifera* (drone brood)	[73,74]
Total phenolic and flavonoid compounds, mealworm oil, defatted mealworm, peptides	*Tenebrio molitor*	[75,76]
Crude extract	*Euconocephalus* sp.	[60]
Crude extract	*Lethocerus indicus*	[60]
Crude extract	*Patanga succincta*	[60]
**Antibacterial activity**		
*p*-Benzoquinone	*Rhynchophorus ferrugineus*	[77]
Hemolymph, seroin 1, 2, and 3 proteins, cecropin, cecropin B moricin, gloverin, vitellogenin, derived oils, recombinant *Bombyx mori* transferrin (BmTf)	*Bombyx mori*	[78,79]
Crude extract	*Oecophylla smaragdina*	[71]
Immunized *Tenebrio molitor* larvae (iTME)	*Tenebrio molitor* larvae	[80]
Mastoparan peptides	*Vespa affinis*	[81]
**Anti-inflammatory activity**		
Protein/peptide hydrolysates, hemolymph, fibroin peptide	*Bombyx mori*	[78,82,83]
Protein/peptide hydrolysates	*Gryllodes sigillatus *, and *Tenebrio molitor*	[76]
Locust cyclopeptides (LCPs)	*Locusta migratoria*	
Unknown	*Vespa affinis*	[84]
Glycosaminoglycan	*Gryllus bimaculatus*	[85]
Mealworm oil, defatted mealworm, peptides	*Tenebrio molitor*	[86]
**Anti-collagenase activity**		
Crude extract	*Acheta domesticus*	[60]
Crude extract	*Bombyx mori*	[60]
Crude extract	*Patanga succincta*	[60]
Crude extract	*Euconocephalus* sp.	[60]
Crude extract	*Lethocerus indicus*	[60]
Crude extract	*Omphisa fuscidentalis*	[60]
**Elastase-inhibitory activity**		
Crude extract	*Acheta domesticus*	[60,62]
Crude extract	*Bombyx mori*	[60]
Crude extract	*Euconocephalus* sp.	[60]
Crude extract	*Lethocerus indicus*	[60]
Crude extract	*Patanga succincta*	[60]
Peptide hydrolysates	*Gryllodes* *sigillatus*	[76]
**α-Glucosidase-inhibitory activity**		
Protein/peptide hydrolysates	*Bombyx mori, Tenebrio molitor*	[76]
**Hepatoprotective activity**		
Peptide (AGLQFPVGR)	*Allomyrina dichotoma*	[64]
Peptide (LE, AKKHKE)	*Tenebrio molitor*	[64]
**Inhibitory activity of pancreatic lipase**		
Crude extract	*Tenebrio molitor*	[62]
**Antidiabetic/insulin-like/insulin-like peptide (ApILP)**		
Sericin (green cocoon shell)	*Bombyx mori*	[87]
**Antidiabetic**		
Unknown	*Apis mellifera* (bee tea)	[72]
Peptides (EIAQDFKTDL)	*Allomyrina dichotoma*	[64]
Cationic peptide	*Gryllodes sigillatus*	[88]
**Angiotensin-converting enzyme (ACE) inhibition**		
Peptides (ASL, GNPWM)	*Bombyx mori*	[64]
**Immune-enhancing activity**		
Crude extract	*Gryllus bimaculatus*	[89]

## Data Availability

Not applicable.
